# Influence of Electrode Design and Contacting Layers on Performance of Electrolyte Supported SOFC/SOEC Single Cells

**DOI:** 10.3390/ma9110906

**Published:** 2016-11-08

**Authors:** Mihails Kusnezoff, Nikolai Trofimenko, Martin Müller, Alexander Michaelis

**Affiliations:** Fraunhofer Institute for Ceramic Technologies and Systems (IKTS), Winterbergstr. 28, Dresden 01277, Germany; nikolai.trofimenko@ikts.fraunhofer.de (N.T.); martin.mueller@ikts.fraunhofer.de (M.M.); alexander.michaelis@ikts.fraunhofer.de (A.M.)

**Keywords:** solid oxide fuel cell, MEA, electrolyte supported cell

## Abstract

The solid oxide cell is a basis for highly efficient and reversible electrochemical energy conversion. A single cell based on a planar electrolyte substrate as support (ESC) is often utilized for SOFC/SOEC stack manufacturing and fulfills necessary requirements for application in small, medium and large scale fuel cell and electrolysis systems. Thickness of the electrolyte substrate, and its ionic conductivity limits the power density of the ESC. To improve the performance of this cell type in SOFC/SOEC mode, alternative fuel electrodes, on the basis of Ni/CGO as well as electrolytes with reduced thickness, have been applied. Furthermore, different interlayers on the air side have been tested to avoid the electrode delamination and to reduce the cell degradation in electrolysis mode. Finally, the influence of the contacting layer on cell performance, especially for cells with an ultrathin electrolyte and thin electrode layers, has been investigated. It has been found that Ni/CGO outperform traditional Ni/8YSZ electrodes and the introduction of a ScSZ interlayer substantially reduces the degradation rate of ESC in electrolysis mode. Furthermore, it was demonstrated that, for thin electrodes, the application of contacting layers with good conductivity and adhesion to current collectors improves performance significantly.

## 1. Introduction

The electrolyte supported planar solid oxide fuel cell (ESC) is the most widespread cell type utilized in commercial SOFC stacks/systems. The long-term, redox and thermal cycling stability of electrolyte supported cells based on 3 mole % Y_2_O_3_ stabilized ZrO_2_ (3YSZ) [1], 6 mole % Sc_2_O_3_ stabilized ZrO_2_ (6ScSZ) [2] and 10 mole % Sc_2_O_3_ stabilized ZrO_2_ with 1 mole % CeO_2_ addition (10Sc1CeSZ) [3] has been demonstrated and improved in the course of continuous development; meanwhile, cost-effective ESC is available as a commercial product [4,5]. Developed cells currently satisfy major requirements (thermal cycling, accident events such as load throw-off, abrupt cooling down and anode oxidation) for use in robust SOFC systems and are used in SOFC stacks of Bloom Energy, sunfire, Hexis and mPower. Although significant gain in cell performance has been achieved in recent years [4,6,7], there is still considerable opportunity for further improvement through the optimization of materials and the microstructure of electrodes, combined with an engineering focus on the volume manufacturing processes (screen printing, co-firing etc.), reproducibility and cost reduction. The utilization of ESCs for solid oxide electrolysis and reversible SOFC/SOEC (Solid Oxide Electrolysis Cell) operation [8] generates further demand for electrode optimization for performance durability.

The total resistance of the membrane-electrode-assembly consists of the electrolyte resistance (ohmic loss arising from the ionic resistance in the solid electrolyte) and the polarization resistances of the air and fuel electrode. The measured absolute resistance value is normed to the electrode area providing a specific characteristic called “area specific resistance” (ASR). This characteristic, corrected to the fuel utilization [9], provides a measure for comparison of different cell types regarding their ability for efficient conversion of chemical energy of fuel to electricity.

The ASR of the electrolyte supported cell type depends strongly on ohmic losses caused by the electrolyte ionic conductivity of a relatively thick (50 μm–210 μm) electrolyte support and its thickness and partially by current collection in thin electrodes. For the reduction of polarization resistance of the electrodes, there are various options from the point of view of electrode materials insofar as there is no demand for densification of the electrolyte and there is some flexibility in terms of sintering temperature in the range of 1200 °C–1350 °C.

In this paper, we provide an overview of our work performed on the optimization of ESC electrodes and contacting layers for SOFC/SOEC operation.

## 2. Results and Discussion

Traditionally, a single SOFC cell has a Ni/8YSZ (8 mole % Y_2_O_3_ stabilized ZrO_2_) functional layer as a fuel electrode, where yttria stabilized zirconia was used due to good chemical compatibility to ZrO_2_-based electrolyte in comparison to doped ceria [10,11]. However, the reduction of sintering temperature allows the interaction between ceria and doped zirconia described in [10,11] to be overcome and the substitution of NiO/8YSZ by the NiO/CGO fuel electrode becomes possible. The enhancement of cell properties for operation in fuel cell and electrolysis mode; the reduction of contacting losses by the optimization of the fuel and air electrode; and the enhancement of cell performance utilizing contacting layers is described in the following sections.

### 2.1. Influence of Anode Composition on ESC Performance

#### 2.1.1. Performance under Standard SOFC Conditions

**Figure 1:**
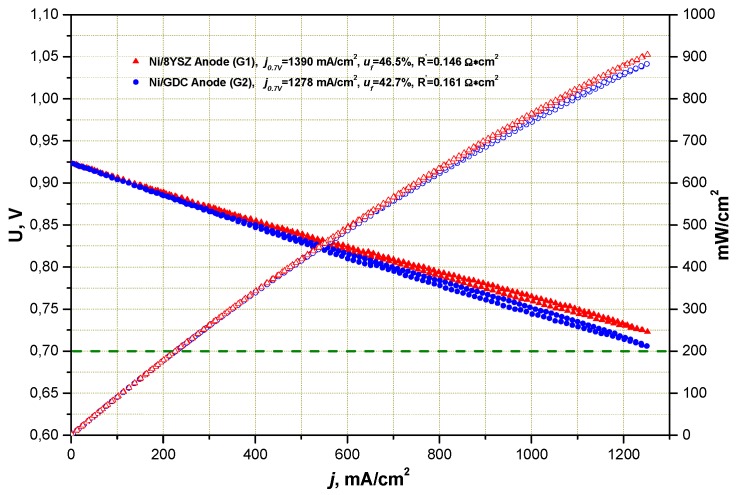
I–V characteristics of single cells with Ni/8YSZ and Ni/CGO electrodes at 850 °C in H_2_:H_2_O = 50:50 (R-values correspond to area specific resistance (ASR) corrected to the fuel utilization).

**Figure 2:**
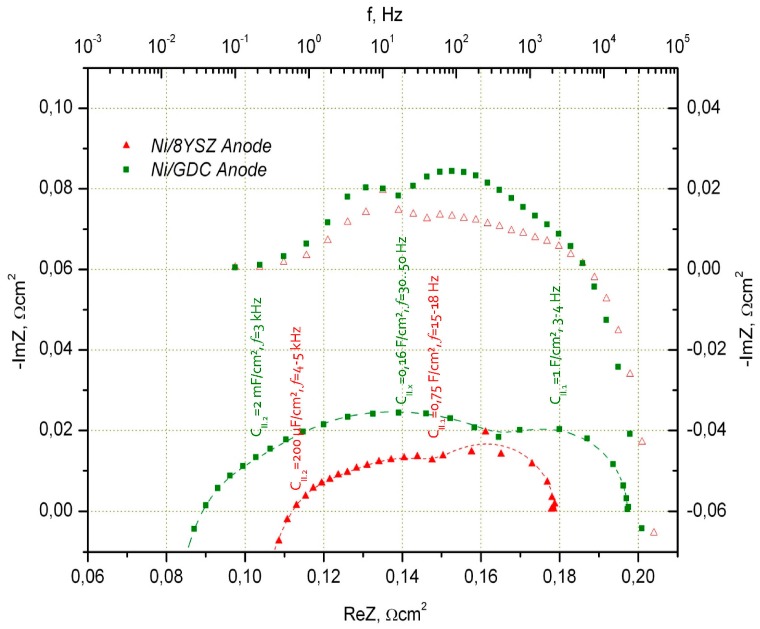
Impedance spectra of MEAs with the LSMM’/10Sc1CeSZ cathode and the Ni/YSZ and Ni/CGO anode at 850 °C with air as oxidant and H_2_:H_2_O = 50:50 as fuel.

**Figure 3:**
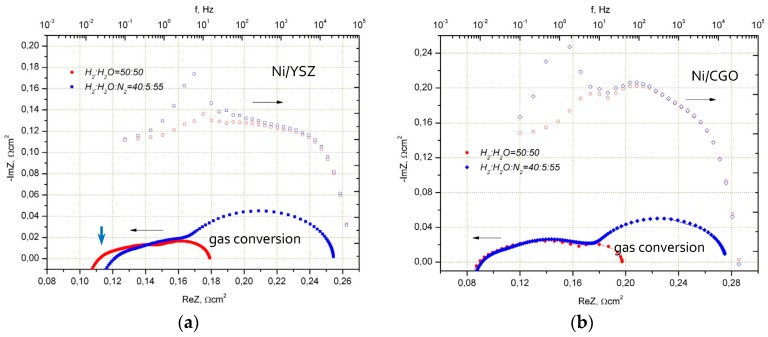
Impedance spectra of MEAs with the LSMM’/10Sc1CeSZ air electrode and the Ni/YSZ (**a**) and Ni/CGO (**b**) fuel electrode at 850 °C with air as oxidant in fuel with different steam content.

Performance of ESC on the basis of a 10Sc1CeSZ substrate with a thickness of 150 μm with a Ni/8YSZ and Ni/CGO fuel electrode is shown in Figure 1. It is well observed that, under ideal operating conditions (H_2_:H_2_O = 50:50), there is no strong deviation in I–V characteristics between both cell types. Impedance spectra in Figure 2 and Figure 3 show the differences of two cells more clearly. A more detailed analysis of cell performance by variation of fuel gas composition (comparison of H_2_:H_2_O = 50:50 to N_2_:H_2_:H_2_O = 55:40:5 in Figure 3) shows that in low humidified fuel the resistance of the cell with Ni/8YSZ mode increases in comparison to the Ni/CGO containing cell, where the polarization resistance of electrodes stays constant.

The deconvolution of spectra was made using the equivalent circuit described in Chapter 3.

It was found that the reaction on the Ni/8YSZ anode can be described using two arcs: (i) an arc for dissociative hydrogen adsorption, which takes place in one step (low frequency arc II.1 in Figure 2, *f* = 15 Hz–18 Hz) and a quick charge transfer reaction at the Ni/8YSZ interface (high frequency arc II.2 in Figure 2, *f* = 4 kHz–5 kHz). For the low frequency arc, we obtained the capacitance in the range of 750 mF/cm^2^ (which is a reasonable value for electrochemical capacitance of metallic surfaces and can be a product of hydrogen adsorption on the active sites on Ni). For the high frequency arc, we observe a capacitance of C_II.2_ = 200 μF/cm^2^ for Ni/YSZ blocking ionic/electronic interface. C_II.2_ grows by increasing the operating temperature of the cell and the low frequency arc capacitance stays more or less independent from the temperature.

In the Ni/CGO electrode, some differences to the Ni/YSZ anode were observed:

(i) High frequency arc (3 kHz–7 kHz) capacitance had higher values compared to the Ni/8YSZ anode (ca. 2 mF/cm^2^)
(ii) Low frequency arc (ca. 4 Hz–5 Hz) capacitance is also higher compared to Ni/8YSZ and is in the range of 1 F/cm^2^ (low frequency adsorption impedance in the arc of air (LSM-based) electrode appears at similar frequencies and the air and Ni/CGO fuel electrode arcs are difficult to separate under selected measurement conditions)
(iii) Additional electrochemical process with capacitance of 160 mF/cm^2^ at 30 Hz–50 Hz is found. This value was not dependent on temperature and H_2_O content in the fuel (5%–50%).

The additional peak in the Ni/CGO impedance spectrum is associated with oxygen capacity of ceria connected with a change of oxygen stoichiometry. The capacity values for this peak (C_II,x_) are in good agreement with the values estimated for CGO particles, considering them as oxygen storage with capacity defined by their stoichiometry.

Figure 3 shows the performance of the Ni/8YSZ and Ni/CGO fuel electrodes in fuel with lower steam content (H_2_:H_2_O = 40:5) in comparison to their operation in strongly humidified hydrogen (H_2_:H_2_O = 50:50). It is seen that the Ni/8YSZ electrode impedance is sensitive to reduction of steam content at high frequencies. As far as electrolyte resistance is independent from pO_2_ for H_2_/H_2_O ratios used in our work, the high frequency polarization arc (*f* kHz = 4 kHz–8 kHz) must be responsible for observed changes.

On the contrary, Ni/CGO fuel electrode impedance stays constant even in fuel with lower steam content. In both electrodes at a steam content of 5%, similar gas conversion impedance at low frequencies was observed.

**Figure 4:**
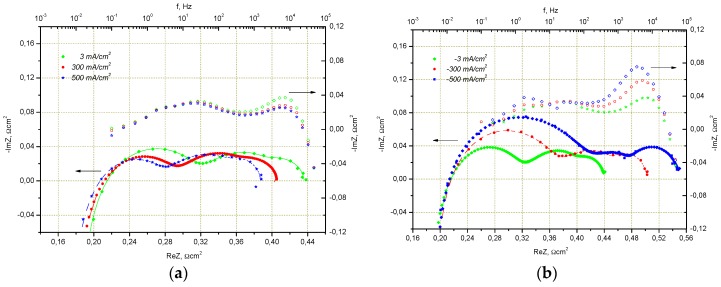

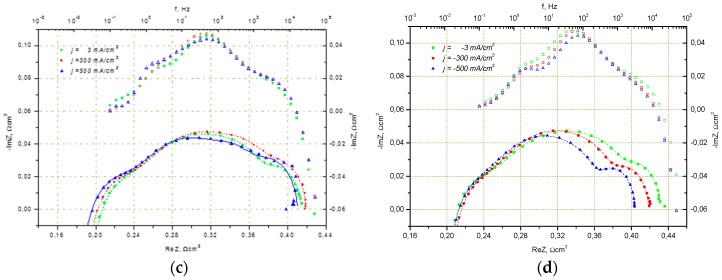
Impedance spectra of electrolyte supported cell (ESC) with LSMM’/10Sc1CeSZ and Ni/YSZ electrodes at 800 °C in the fuel cell (**a**) and electrolysis; (**b**) modes (air, H_2_/H_2_O = 50/50) at different current densities and impedance spectra of the ESC with LSMM’/ScSZ and Ni/GDC electrodes at 800 °C in the fuel cell; (**c**) and electrolysis; (**d**) modes (air, H_2_O/H_2_O = 50/50) at different current densities.

A change of impedance spectra of cells with the Ni/8YSZ and Ni/GDC electrodes under constant current is shown in Figure 4. Anodic polarization (current flow generates additional steam on the fuel side) typical for cell operation in SOFC mode reduces the total impedance of the Ni/8YSZ electrode however the impedance of the Ni/CGO electrode under the same conditions stays unchanged. This observation is in good agreement with the changes seen in the impedance spectra in Figure 3, by increasing the steam content in hydrogen. The cathodic polarization of Ni/8YSZ and Ni/GDC under electrolysis conditions results in an increase of total impedance for both cells. Although the total impedance of the Ni/CGO electrode undergoes small changes only, the impedance of the Ni/8YSZ electrode grows by 25% of the initial value at open circuit voltage (OCV), increasing current density from 0 mA/m^2^ to 600 mA/m^2^.

Based on these observations, we suppose that the mechanism of electrochemical reaction in Ni/CGO is different from that in Ni/8YSZ. The CGO surface is probably strongly involved in the electrochemical process and even substitutes Ni active sites, reducing their function to a collection of electrons generated by electrochemical reactions from the CGO catalyst. This assumption is also supported by the observation of a better sulphur tolerance of new Ni/CGO anodes by Kavurucu-Schubert [12] which could be only explained by ceria surface involvement into hydrogen oxidation.

Under reducing conditions (pO_2_ = 10^−12^ bar–10^−14^ bar), fully stabilized ceria undergoes transformation to a mixed ionic/electronic conductor [13] because of the valence change of Cerium from Ce^4+^ to Ce^3+^. The valence change is compensated by oxygen loss from the lattice and an increase of the oxygen vacancy concentration, creating electrons to keep its electro-neutrality. The additional electrons raise the n-type conductivity of CGO (~pO_2_^−1/4^), however, the ionic conductivity stays constant up to the pO_2_ = 10^−14^ bar at temperatures below 900 °C [14]. In this state, ceria is catalytically active for redox processes, contrary to purely ionic conductive stabilized zirconia insofar as it can easily transport the electrons necessary for electrochemical reactions.

The absolute value of polarization resistance of the Ni/CGO fuel electrode was, nevertheless, initially higher compared to Ni/8YSZ (see Figure 2 and Figure 3). For this reason, different CGO powders were tested for paste preparation and a Ni/CGO fuel electrode with reduced polarization resistance was obtained. The characteristics of the optimized ESC, developed as result of the substitution of Ni/8YSZ to Ni/CGO (optimized anode in 3rd cell generation (G3 cell)), are summarized in Table 1. The resistance of the G3 cell is similar to that of the Ni/8YSZ based cell (0.18 Ω·cm^2^, see Figure 2 and Figure 3). The G3 cell electrodes have been used as a basis for further improvements for long-term stable electrolysis operation and for the development of cells with ultrathin (<60 μm) 3YSZ electrolytes.

#### 2.1.2. Electrode Optimization for Performance in SOEC Mode

Several reasons for degradation have been observed and discussed in the literature for the Ni based fuel electrode, as well as for the oxygen electrode during operation in SOFC/SOEC mode at high temperature and at high current densities [15,16,17]. In particular, the formation of oxygen bubbles at the grain boundaries and the delamination of the oxygen electrode under SOEC conditions [18,19] connected with related phenomena, has been tried to be prevented by using a mixed ionic/electronic conductor such as La_0.6_Sr_0.4_Co_0.2_Fe_0.8_O_3-x_ (LSCF). We have tried an alternative solution and used the additional porous interlayer between the support and multilayer oxygen electrode to prevent the pressure accumulation in closed pores at the interface which facilitates the delamination.

To study the influence of the interface nature, cells with different interlayers (first layer in the multilayer structure of the air electrode; A—8YSZ; B—ScSZ; C—SDC (Sm_2_O_3_ fully stabilized CeO_2_) are measured and compared under the same experimental conditions.

**Figure 5:**
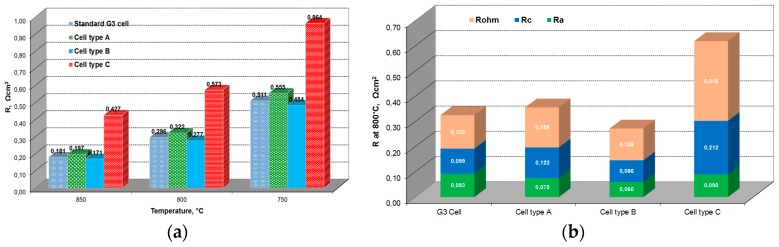
Total area specific cell resistance (**a**) vs. operating temperature and constituents of ASR; (**b**) obtained by the deconvolution of impedance spectra of the cells with different interlayers measured at 800 °C in air: H_2_/H_2_O mixture (50/50) at j = 300 mA/cm^2^ in fuel cell mode (without correction to fuel utilization).

The ASR values of studied cells at different temperatures are shown in Figure 5. The ASR of the G3 cell without an additional layer is lower (~10%) than the value for the cell with an 8YSZ layer and ~2 times lower in comparison to the cell with an SDC layer. Only the cell with a ScSZ interlayer shows better electrochemical performance than the unmodified G3 cell. The deconvolution of impedance spectra at different temperatures clearly shows the decrease of the cathodic and anodic polarization resistance with similar ohmic resistances for the cell type B in comparison with the standard G3 cell.

The introduction of the porous interlayer, based on 8YSZ, results in additional ohmic resistance. The resistance of such a layer substantially increases the total ohmic losses due to its lower conductivity connected with low sintering temperature and open porosity.

The highest ohmic resistance (0.316 Ω·cm^2^) was measured for the cell with a SDC layer. The reason for such a high resistance is probably the formation of intermediate phases with low ionic conductivity during co-firing at the interface 10Sc1CeSZ/SDC and SDC interaction with perovskite at the relatively high sintering temperature used for co-firing.

Based on these results, the cells type A and B were used for further investigation under SOFC and SOEC conditions.

**Figure 6:**
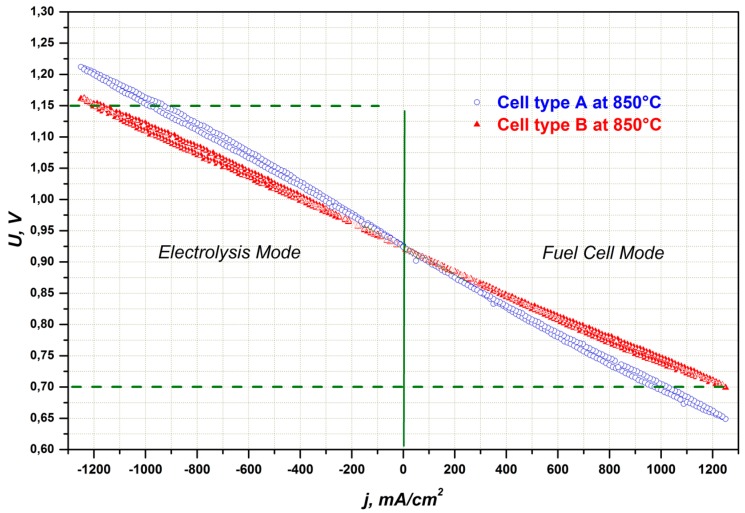
I–V characteristics of cells with 8YSZ (type A) and ScSZ (type B) interlayers measured in both electrolysis and fuel cell mode at different temperatures under H_2_/H_2_O = 50/50.

Figure 6 shows I–V curves for cell types A and B under 50% humidity at 850 °C and 800 °C. The slope of the I–V curve of the cell with an additional layer, based on ScSZ, is lower than that of the cell based on 8YSZ. The area-specific resistance (ASR) corrected to the fuel utilization, calculated for cell type B at 800 °C, is 0.262 Ω·cm^2^ and lower than the ASR value of cell type A.

The cells were exposed to the galvanostatic electrolysis conditions with different current densities and different temperatures for examination of performance durability. The evolution of the cell voltage and calculated ASR as a function of time is summarized in Table 2.

The voltage of the cells with interlayers A and B increased with the time during operation in electrolysis mode. The rate of observed degradation depends mostly on used materials (8YSZ or ScSZ) and is nearly independent from the applied current density used (up to −500 mA/cm^2^). Both cells A and B showed different degradation rates at −300 mA/cm^2^ at 850 °C. Cell A started with a cell voltage of 952 mV, which is in good agreement with the I–V-curves shown in Figure 5. Within the first 200 h, the voltage of cell A increased 12 mV and afterwards some voltage decrease was observed until the end of the experiment. The overall estimated degradation rate was 8 mV/1000 h or 31 mΩ·cm^2^/1000 h respectively.

The voltage of the cell with interlayer B increased linearly without any activation period, as is the case for cell A, but the degradation rate was similar for the same experimental conditions (31 mΩ·cm^2^/1000 h).

For both cell types, the initial degradation phase was more pronounced if the temperature was decreased from 850 °C to 800 °C. After 200 h, the operating voltage of cell B at −500 mA/cm^2^ at 800 °C continued to increase in a close-to-linear manner, achieving a constant value after more than 1000 h of operation. The total increase of cell voltage was only 33 mV over 5200 h. The estimated degradation rate was 14 mΩ·cm^2^/1000 h, twice as low as those of both cell types at 850 °C.

The difference in the degradation behavior of cell types A and B using 8YSZ and ScSZ interlayers could not be explained, even after examination of interfaces in SEM. No air electrode delamination was found for all types of tested cells.

The electrode deactivation during the first 200 h of operation in SOEC mode was attributed to the fuel electrode. We suppose that this behavior is connected with H_2_O concentration polarization and the diffusion of H_2_O towards three phase boundaries. Further investigations are necessary to clarify this phenomena.

### 2.2. Influence of Contacting

3YSZ electrolytes with a thickness of 90 μm are often used as a substrate for ESC because of their outstanding mechanical properties and low cost. G3 electrodes, developed for 165 μm 10sc1CeSZ substrates, could be easily adapted to 90 μm 3YSZ. As far as 3YSZ substrates have higher resistance due to lower ionic conductivity of partially stabilized zirconia, a different approach for the reduction of ohmic losses such as reduction of electrolyte thickness up to 50 μm [20] should be implemented. The reduction of electrolyte thickness up to 50 μm with conventional G3 electrodes resulted in higher warpage of the cell during electrode co-firing. To minimize the warpage, the electrode thickness has to be reduced and the paste shrinkage behavior optimized. In particular, the reduction of electrode thickness leads to lower warpage and increases in-plane resistance for current collection (especially in the air electrode). To compensate for these additional losses, contacting layers (CCL = Cathode (air electrode) Contacting Layer) made of materials with high conductivity and a sintering temperature of 870 °C–930 °C were applied.

**Figure 7:**
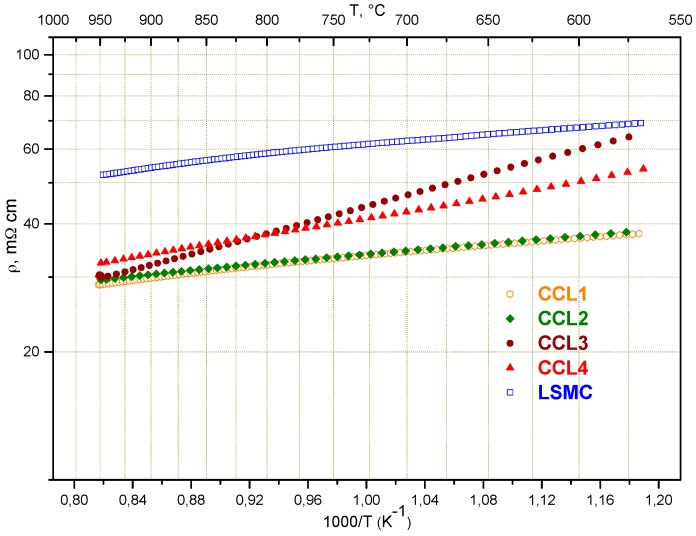
Resistance of different CCL layers in dependence of temperature.

The lanthanum strontium cobaltite manganite perovskite (LSMC), FeCoMn oxide spinel MCF, and CuNiMn oxide spinel CNM are widely used to contact air electrodes and interconnects. These materials show good electrical conductivity at an operating temperature above 700 °C. Figure 7 presents the temperature dependence of resistance of the screen printed CCL1–4 layers and pure LSMC. The resistance decrease for CCL1 and CCL2 compositions in comparison with pure LSMC can be explained by the improvement of morphology of the layer, and its higher density and homogeneity, due to the introduction of the sintering additives. It should be mentioned that all studied samples were sintered at a relatively low temperature and the presented resistance data were not corrected on porosity. The comparison of the obtained data allows the evaluation of all studied CCLs according to their resistance at 850 °C as follows: CCL1 ≈ CCL2 < CCL3 ≈ CCL4 < LSMC. The layers have been applied on the surface of the 3YSZ based MEAs, the performance of which was measured and the ASR was calculated.

Data of ASR (corrected for fuel utilization) for cells based on thin 3YSZ presented in Table 3 are in good agreement with resistance data from Figure 6. Except the cell, which was coated with CCL4 based on CuNiMn oxide spinel (CNM), all others show performance which correlates with the conductivity data of the corresponding contacting material.

A clear decrease of ASR (more than −60 mΩ·cm^2^) with screen printed CCLs was observed. The decrease of ASR for CCL4 is even more significant (−95 mΩ·cm^2^) compared with other samples. We suppose that the CCL4 layer provided better adhesion to the current collector meshes during cell initialization which probably resulted in better contact (larger number of contact pints) between the current collector mesh and cell surface.

**Figure 8:**
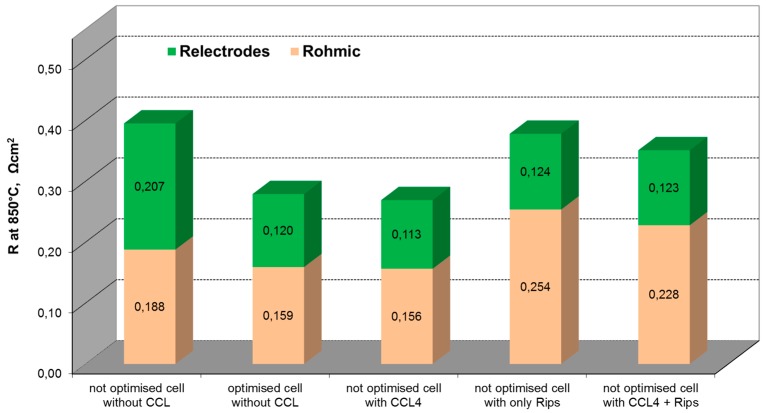
Constituents of ASR obtained by deconvolution of impedance spectra of cells with different CCLs, measured at 850 °C at 300 mA/cm^2^ (the ASR of the cells, resulted from impedance spectroscopy, was not corrected to fuel utilization).

Based on these results and using CCL4 as the screen printed contacting layer, the comparison of the ASR of the 3YSZ based cell without CCL; with CCL; with CCL and contacting ribs; and with contacting ribs only (see Figure 8 for details) was done.

As illustrated in Figure 8, in the presence of CCL between the cathode and ribs, the ASR of the cell is decreased (ΔR = 94 mΩ·cm^2^ for the not optimized cell). The results indicate that the contact layer is a significant factor in the electrical performance of the single cells. Based on these results, the further optimization of the rib’s design can be done using well-proven (since 2007 in use at Fraunhofer IKTS) simulation tools describing the oxygen concentration profile under ribs as a function of current density and ohmic losses due to lateral current transport between the ribs followed by experimental verification.

## 3. Materials and Methods

The electrolyte supported MEAs on dense 3YSZ and 10Sc1CeSZ substrates (50 mm × 50 mm × (0.05 mm–0.165 mm)) with screen-printed nickel oxide and the yttria stabilized zirconia cermet fuel electrode (NiO/8YSZ), the nickel oxide and fully gadolinia (Gd_2_O_3_) stabilized ceria (CeO_2_) cermet fuel electrode (NiO/CGO) and the co-doped lanthanum strontium manganite (La_1−x_Sr_x_Mn_1−y_M’_y_O_3−δ_) and scandia stabilized zirconia (ScSZ) composite air electrode were sintered in co-firing at temperatures <1325 °C. The lanthanum strontium manganite, with an additional transition metal on the B-site La_1−x_Sr_x_Mn_1−y_M’_y_O_3−δ_ (LSMM´), Sc_2_O_3_ stabilized ZrO_2_ (ScSZ), gadolinia stabilized ceria (CGO) and the NiO powders used in this work were supplied by different manufacturers, according to the specifications for the stoichiometry, crystalline phase, specific surface and particle size distributions.

The electrode pastes were prepared using the equipment and steps described in [3,21,22] which have been significantly modified and evaluated for up-scaling. Additional wetting agents were used to improve screen printing characteristics such as thickness uniformity, which are very important for large area prints required for cathode and anode application.

The electrochemical experiments were carried out on the cells with symmetrically screen-printed air and fuel electrodes with lateral dimensions of 40 × 40 mm^2^. A multilayer fuel electrode (three layers) and a two-layer air electrode were used.

The first air electrode layer (~20 μm) was composed of a mixture of lanthanum strontium manganite with additional transition metal on B-site LSMM´ and ScSZ in proportion ca. 50:50 vol %. The second layer (~25 μm) consisted of LSMM´ and had a current collector function. Porosity of the sintered layers is 25%–35%.

The first anode layer can be mainly composed of CGO and helps for the adhesion of the upper anode layers to the substrate. The next anode layers are more electrochemically active and the upper anode layer with the highest volume content of NiO is a current collector. The electrochemically active anode layer has a porosity of 30%–40% after NiO reduction to Ni before the start of cell operation. The total anode thickness was <35 μm. The electrodes were sintered in co-firing at temperatures <1325 °C.

**Figure 9:**
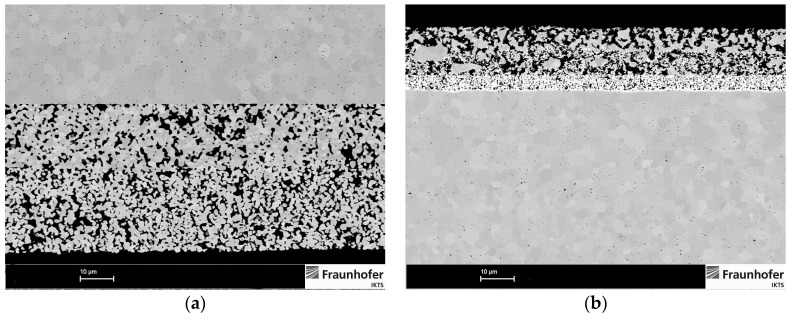
Microstructure of the bi-layer LSMM’/10Sc1CeSZ air electrode (**a**) and the multilayer Ni/CGO fuel electrode; (**b**) after reduction in hydrogen before starting the cell operation.

The morphology of the studied electrodes was analyzed using field emission scanning electron microscopy and is shown in Figure 9. An air electrode consisting of an electrochemically active layer (composite of perovskite and ScSZ) and current collecting layer (perovskite only) is shown in Figure 9a. The fuel electrode in the reduced state, consisting of an adhesive layer, electrochemically active layer and current collecting layer, is shown in Figure 9b. The three phase boundary length per 1 cm^2^ of the electrode area of the electrodes has not been determined.

The cells were first heated up to 930 °C in air/nitrogen and then reduced using a hydrogen/nitrogen mixture. The air flow of 60 sl/h and hydrogen:steam:nitrogen or hydrogen:steam flow of 40 sl/h are fed to the cell, resulting in the open circuit voltage which corresponded well to its theoretical value at an elevated temperature. The current–voltage characteristics are measured at different temperatures to validate the cell performance.

**Figure 10:**
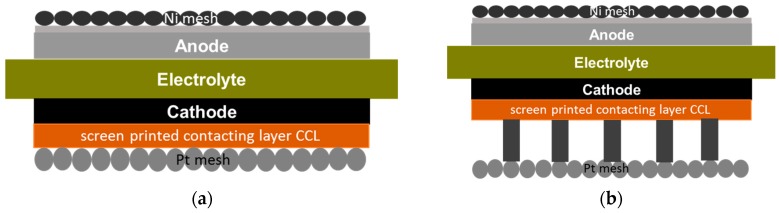
Evaluation of cell and CCL resistances (**a**) and simulation of stack situation (**b**).

Electrochemical investigations on cells were carried out using the advanced test bench for cell characterization developed at Fraunhofer IKTS (Dresden, Germany), as well as in an Evaluator C50-HT test bench developed by FuelCon AG (Barleben, Germany). The apparatus consists of a custom-built ceramic housing integrated in a furnace operated by a temperature controller (Eurotherm, West Sussex, UK) enabling the temperature–time profile management up to 1000 °C. Pt and Ni meshes were used as the contact material for the cathode and anode respectively. To vary the contact between the cathode and the Pt mesh of the current collector, the cathode of the sintered cell is coated with contact paste (CCL = Cathode (air electrode) Contacting Layer) by screen printing (Figure 10a). For practical application, however, during assembling, the SOFC stacks the small channels, which are usually formed from CCL or interconnect material and are commonly used to carry the air gas flow. Such “ribs”, which define the flow channels and make direct contact between the interconnect and the cathode, were realized by stencil printing (Figure 10b).

As a potential contact layer, the lanthanum strontium cobaltite manganite perovskite (LSMC) with different sintering additives (CCL1 and CCL2), the mixture of LSMC and MnCoFe oxide spinel MCF (CCL3) as well as CuNiMn oxide spinel CNM (CCL4) were used.

The conductivity of the CCL used for the cathode side contacting was measured using a DC (direct current) four-point probe method. Pastes were prepared from powders and screen-printed as single stripes (30 mm × 2 mm) onto 8YSZ substrates; four screen printed gold contacts were applied for electrical contacting. The samples were sintered at 950 °C for 3 h. The thickness of the layers was between 20 μm to 40 μm after sintering. The temperature dependence of the conductivity was measured in the range of 500 °C to 950 °C.

**Figure 11:**
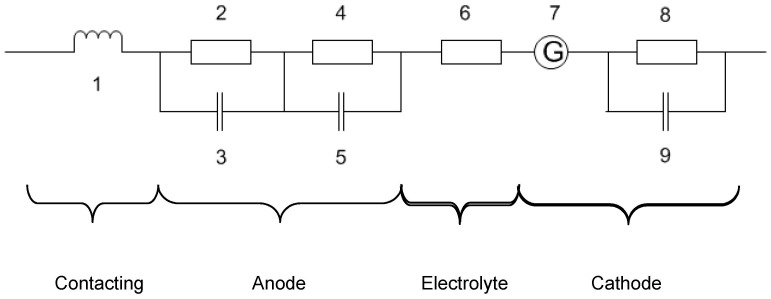
Equivalent circuit for calculation of MEA impedance.

The developed cells were characterized by impedance spectroscopy under the current load (up to 650 mA/cm^2^ at SOFC and up to 500 mA/cm^2^ at SOEC conditions) at temperatures of 700 °C–950 °C in an air:hydrogen/steam dual atmosphere using impedance analyzer IM6 (Zahner, Germany). The frequency was varied between 10 mHz and 100 kHz, the excitation AC voltage was fixed at 10 mV. The constituents of the anode, cathode and electrolyte in the overall resistance are extracted from impedance spectra using Thales^®^ Software (Zahner, Germany) and an equivalent circuit shown in Figure 11. Polarization resistance of the electrodes obtained from impedance spectroscopy was normed to their lateral dimensions (40 × 40 mm^2^ = 16 cm^2^) and plotted in Nyquist and Bode diagrams.

The equivalent circuit of a solid oxide fuel cell can be described as a combination of inductors, capacitors and resistors. Following equivalent circuit for fitting the impedance spectra of the MEA was used.

Basically, the circuit consists of one inductor for the contacting of the cell (1), three charge capacitance elements (3,5,9) and four resistance elements (2,4,6,8). Elements 2 and 3 represent the dissociative hydrogen adsorption on the catalyst surface, the fourth and fifth elements represent the charge transfer at the interface. Element 6 represents resistance due to the ionic conductivity of the electrolyte which should follow the Ohm’s law. The eighth and ninth elements represent the adsorption of oxygen on the catalyst surface, the seventh element is the Gerisher element and represents a diffusion/charge transfer at three phase boundary (TPB) (air side).

The current–voltage characteristics with a current rate of 0.2 A/min up to 20 A and impedance spectra have been measured at different experimental conditions. The value of the total ASR of the cell was obtained from an operation at a fixed current density and corrected to fuel utilization. Total cell resistance obtained from impedance spectra (impedance value at frequency 10 mHz) has not been corrected for fuel utilization.

The long-term stability test is performed with a constant current density, in ceramic housing, in an appropriate air/H_2_:H_2_O:N_2_ dual atmosphere. The cells were first heated up to 930 °C in nitrogen, then reduced and activated at 300 mA/cm^2^ in SOFC mode. Afterwards, the cell was cooled down to operation temperature and tested under the desired conditions. During the long-term operation, the current load throw-offs, thermal cycles and redox cycles took place and became part of the experiment. The measurement of the degradation—the change in the cell voltage at constant current during the overall operation of the cell—was calculated from a linear fit of a written voltage–time dependence [3].

## 4. Conclusions

Performance and stability of electrolyte supported cells for operation in SOFC and SOEC modes was significantly enhanced by the substitution of the traditional Ni/8YSZ fuel electrode with Ni/GDC. The reason for this improvement is the utilization of CGO as a catalyst for electrochemical hydrogen conversion, which is much more stable and less sensitive to impurities than nickel.

The introduction of a porous interlayer on the air side is an effective option to stabilize and use the LSM-based electrodes for electrolysis operation. A ScSZ based interlayer even had no effect on overall cell resistance, providing better stability by the same ASR as a reference cell.

The ASR of the electrolyte supported cell can be effectively reduced by the reduction of electrolyte thickness. To keep the cell planarity, the electrode thickness should also be reduced. Ohmic losses for current collection in electrodes with reduced thickness can be compensated for by contacting layers. It was shown that better conductivity of the contacting material, as well as its contact to the current collector, leads to lower ASR of such cells.

## Figures and Tables

**Table 1:** ASR (with correction to fuel utilization) of G3 electrolyte supported cells with the optimized Ni/CGO anode based on 165 μm 10Sc1CeSZ with scattering (∆ASR) observed on different cells.

Temperature (°C)	ASR (Ω·cm^2^)	∆ASR (Ω·cm^2^)	Voltage (mV) at 300 mA/cm^2^ in H_2_/H_2_O/N_2_ = 40/5/55
850	0.178	±0.010	935
800	0.286	±0.013	915
750	0.508	±0.019	865
700	0.918	±0.036	745

**Table 2:** Changes of cell voltage and ASR for different cells during the long-term galvanostatic operation in electrolysis mode in a H_2_/H_2_O mixture with a molar ratio of 20/80.

Cell Type	Current Density (mA/cm^2^)	Temperature (°C)	Testing Period (h)	Measured Voltage (mV)			ASR (Ω·cm^2^)		
				Initial	After 200 h	Final	Initial	After 200 h	Final
A	−300	850	1370	952	964	963	0.336	0.381	0.378
B	−300	850	1270	917	918	929	0.224	0.229	0.264
B	−500	850	1180	962	963	974	0.238	0.240	0.264
B	−500	800	5200	1035	1043	1068	0.340	0.360	0.413

**Table 3:** ASR of G3 electrolyte supported cells based on thin 3YSZ (electrolyte thickness of 50 μm) measured at 300 mA/cm^2^ in air/H_2_:H_2_O mixture (50:50) without and with different CCLs.

CCL	Temperature (°C)	ASR (Ω·cm^2^)	Working Voltage (mV) (at 300 mA/cm^2^ in H_2_:H_2_O:N_2_ = 40:5:55)
no	850	0.359	878
CCL1	850	0.292	898
CCL2	850	0.287	898
CCL3	850	0.293	897
CCL4	851	0.264	902
